# Molecular detection of *Hepatozoon felis* in cats from Maio Island, Republic of Cape Verde and global distribution of feline hepatozoonosis

**DOI:** 10.1186/s13071-019-3551-3

**Published:** 2019-06-11

**Authors:** Cristina Pereira, João Pedro Maia, Ricardo Marcos, Camilla Luzzago, Pablo Puente-Payo, Paola Dall’Ara, Augusto Faustino, Stefania Lauzi

**Affiliations:** 1 Institute of Biomedical Sciences Abel Salazar, University of Porto, Porto, Portugal; 2 CIBIO Research Centre in Biodiversity and Genetic Resources, InBIO, Universidade do Porto, Campus Agrário de Vairão, Rua Padre Armando Quintas, Nº 7, Vila do Conde, 4485-661 Vairão, Portugal; 3 Departamento de Biologia, Faculdade de Ciências, Universidade do Porto, Rua do Campo Alegre FC4, 4169-007 Porto, Portugal; 4 Department of Veterinary Medicine, University of Milan, Via Celoria 10, 20133 Milan, Italy; 5 Coordinated Research Center “EpiSoMI”, University of Milan, Milan, Italy; 6 Veterinary Teaching Hospital, University of Milan, Lodi, Italy

**Keywords:** Vector-borne pathogens, Anaplasmosis, Ehrlichiosis, Bartonellosis, Phylogeny, Felid

## Abstract

**Background:**

Vector-borne diseases are emerging worldwide and have an important zoonotic relevance. In the last few years, the interest in vector-borne pathogens in cats has increased. However, studies on feline vector-borne pathogens on tropical islands are lacking. Islands differ from continental countries because they have an enclosed population of animals, with all year presence of the vectors and, most often, without vector control measures. This study focused on the molecular identification and phylogenetic analysis of vector-borne pathogens in autochthonous cats with a mixed indoor–outdoor lifestyle from Maio Island, Cape Verde archipelago.

**Methods:**

Blood samples were collected from 80 asymptomatic cats, representing almost a quarter of the total cat population of the island. The presence of DNA of protozoa of the genus *Hepatozoon* and bacteria belonging to family *Anaplasmataceae* and to genus *Bartonella* was assessed by PCR and phylogenetic analysis was conducted. Statistical analysis was performed to identify risk factors associated with infection. For feline hepatozoonosis, a worldwide dataset of *Hepatozoon felis* sequences retrieved from mammal species and vectors along with *Hepatozoon* spp. sequences retrieved from felids was generated, phylogenetically analyzed and the geographical and host distribution was assessed.

**Results:**

*Hepatozoon felis* genotype I was identified in 12 (15%) cats from Maio Island whereas none of the cats were PCR positive for the other pathogens tested. No significant association of *H. felis* infection with age, sex, location or presence of vectors was observed by statistical analysis in Cape Verde’s cats. Phylogenetic analysis on the worldwide dataset of feline *Hepatozoon* sequences showed two significant distinct clades for *H. felis* genotype I and II. Different geographical distributions were assessed: *H. felis* genotype I was the only genotype found in Africa and has been reported worldwide, with the exception of Japan and Brazil where only *H. felis* genotype II has been reported.

**Conclusions:**

The identification of *H. felis* genotype I in cats in Maio Island highlights the need to further investigate the significance of *H. felis* genotypes and to clarify the epidemiological aspects of this infection.

**Electronic supplementary material:**

The online version of this article (10.1186/s13071-019-3551-3) contains supplementary material, which is available to authorized users.

## Background

Vector-borne pathogens (VBPs) are recognized as agents causing important emerging diseases worldwide in humans and animals and represent an increasing concern for their zoonotic potential [1]. It is widely recognized that companion animals, especially dogs, play an important role in the epidemiology of vector-borne diseases (VBDs) acting as reservoirs and/or sentinels for several human and/or animal VBDs [2].

Numerous studies have been performed on canine VBDs, whereas feline vector-borne infections have been much less investigated, despite cats being ubiquitous companion animals [3]. Previous publications showed that although cats often have an outdoor lifestyle with a high risk of exposure to arthropods and are susceptible to tick bites and tick-borne agents, they appear to be less affected by tick-borne pathogens compared to dogs in geographical areas endemic for VBDs [2, 4]. This may be attributed to peculiar habits and behavior (e.g. grooming) of cats or to natural resistance to these pathogens or their vectors [2, 4]. Furthermore, the relatively limited knowledge about feline VBDs probably reflects a lack of awareness of these pathogens in cats in endemic areas, likely due to the disease manifesting subclinically in most of the infected cats [5]. Therefore, the control of vectors and VBDs in cats is nowadays considered highly relevant [6].

In the last few years, studies on feline VBPs have shown a wide geographical distribution of these pathogens, especially in areas with endemic VBDs in dogs [7–11]. Vector-borne diseases have also been reported in cats living in confined environments, such as small islands [5].

Among pathogens potentially transmitted by ticks in cats, feline hepatozoonosis has been increasingly reported in cats and wild felids and two *H. felis* genotypes have been identified [12]. The exact vectors and routes of transmission of feline hepatozoonosis are not known but transmission by vectors likely plays a key role as for other species of *Hepatozoon* [13].

Vector-borne pathogens transmitted or potentially transmitted by *Rhipicephalus sanguineus* (*sensu lato*), some of them being zoonotic, have been reported in dogs from Santiago and Maio islands of the Archipelago of Cape Verde [14, 15]. To date, *Rh. sanguineus* (*s.l.*) is the only hard tick that has been reported in the Cape Verde archipelago and it has been observed all year long. Moreover, *Ctneocephalides felis* has been reported among the four more common species of fleas that occur in the Cape Verde archipelago [16]. Although cats in the Cape Verde archipelago may be at risk of VBPs, and because vector control measures are not routinely applied to cats, studies on feline VBPs are lacking in this archipelago. To the best of our knowledge, the only study on feline VBPs in the Cape Verde archipelago was limited to an investigation of the presence of *Dirofilaria*, but this pathogen was not identified in cats [17].

A population of 354 cats has been estimated in Maio Island with most cats being privately owned [18]. Considering that tick-borne pathogens have been previously reported in dogs in Cape Verde, the aims of this study were thus to (i) apply microscopical and molecular detection methods to investigate the occurrence of agents of bacteria family *Anaplasmataceae* and genus *Bartonella*, as well as the protozoan genus *Hepatozoon*, in a representative sample size of cats from Maio Island; (ii) assess the phylogenetic relationships among feline pathogens detected in Maio island and feline *Hepatozoon* sequences reported worldwide, analyzing host and geographical distribution; and (iii) identify risk factors associated with infection in cats from Maio Island.

## Methods

### Animals and sample collection

A representative sample size of 80 animals was calculated using WinEpi (http://www.winepi.net) for the detection of pathogens in the cat population, considering the minimum prevalence of tick-borne pathogens (3.5%) detected in the dog population of Maio Island [15]. Cats were included in the study using a non-probabilistic sampling method. Criteria for inclusion were the owner’s consent, autochthonous origin of animals and age ≥ 6 months. All cats were apparently healthy, but detailed clinical examinations were not conducted. The cats lived indoors with variable access to the outdoors and were considered to have a mixed indoor–outdoor lifestyle. Although climatic conditions of Maio Island allow a potential exposure to vectors all year long, owners were not aware of VBPs and no vector control measures had been used in these cats. Animals were sampled during November of 2012 throughout the island and data on age, sex, locality and presence of ticks or fleas were recorded for each cat.

Two milliliters of blood were collected by jugular venipuncture from each cat. Blood was kept in ethylene diamine tetraacetic acid (EDTA) tubes at 4 °C until further processing (up to 12 h). For each animal, four separate 50 µl dots of blood were spotted onto Whatman^®^ filter paper, dried completely, and stored at 4 °C in order to be used later for molecular analysis.

### Microscopical screening

A buffy coat smear (BCS) was performed for each animal, as detailed elsewhere [19]. Buffy coat smears were stained with a Diff-Quik (Hemacolor, Merck, Darmstadt, Germany) and screened using a microscope (Olympus CX31, Olympus, Tokyo, Japan) at 100× magnification for the presence of blood gamonts or morulae infecting neutrophils, monocytes or platelets.

### Molecular and phylogenetic analysis

DNA was extracted from Whatman^®^ papers using a commercial kit, following the manufacturer’s instructions (NucleoSpin Tissue, Macherey-Nagel, Düren, Germany).

Specific published PCR protocols were used to detect DNA of bacteria of the family *Anaplasmataceae* [20], genus *Bartonella* [21] and protozoa of the genus *Hepatozoon* [22]. DNA extracted from *Anaplasma phagocytophilum* and *Hepatozoon canis* infected dogs and from *Bartonella henselae* infected cats were used as positive controls in PCR reactions. A blank control (water sample) was also included in all PCR reactions. Gel-electrophoresis was conducted using 2% agarose gels.

For *Hepatozoon* spp., the positive samples with a PCR product of 631 bp were purified and sequenced using the forward and reverse primers used for DNA amplification [22]. Sequencing was performed using a commercial sequencing facility (Macrogen Inc., Seoul, South Korea). The sequence data were assembled, and manual editing was performed using BioEdit software v.7.0 (freely available at http://www.mbio.ncsu.edu/BioEdit/bioedit.html) and Geneious v.6.1 (Biomatters Ltd., Auckland, New Zealand). The sequences were then compared with those available in GenBank using BLAST (http://blast.ncbi.nlm.nih.gov/Blast.cgi).

Sequences from Cape Verde cats were aligned with representative *H. felis* genotype I, *H. felis* genotype II, *H. canis*, *H. americanum*, *H. silvestris*, *H. martis* sequences retrieved from GenBank, using Clustal X in BioEdit software v.7.0. Phylogeny was estimated by a neighbor-joining algorithm (NJ) [23] and by maximum likelihood (ML) [24] methods with 1000 bootstrap replicates using MEGA v.7 [25].

The representative *H. felis* sequence obtained in this study was deposited in GenBank under accession number MK836092.

### Host and geographical distribution of feline *Hepatozoon* sequences reported worldwide

A dataset of 269 feline *Hepatozoon* sequences retrieved from GenBank was generated. Sequences were retrieved from peer-reviewed journals [5, 7, 12, 13, 26–60] or were publicly available from GenBank (accessed on 18 February 2019). Selection criteria for feline *Hepatozoon* sequences were identification in GenBank as *Hepatozoon felis* (retrieved from mammals and vectors) and *Hepatozoon* spp. sequences reported worldwide in domestic and wild felids (Additional file 1: Table S1). All the feline *Hepatozoon* sequences included in our dataset were phylogenetically analyzed as described above for sequences from cats of Maio Island. Phylogenetic analysis was performed to identify the genotype of *H. felis* for the sequences deposited in GenBank that had not previously been assigned to a genotype.

According to the results of the phylogenetic analysis on the comprehensive dataset of feline *Hepatozoon* sequences and the representative *H. felis* sequence from Maio Island cats, the host and geographical distribution were assessed for each *H. felis* genotype and for other *Hepatozoon* species identified in felids.

### Statistical data analysis

In the presence of negative results for the pathogens tested, the maximum possible prevalence in the total cat population was calculated using WinEpi (http://www.winepi.net).

Pearson’s Chi-square test was used to evaluate the differences between proportions of infected cats and sex, age, village of origin, presence of ticks and presence of fleas. Statistical comparisons were carried out using SPSS v.15.0 software, taking *P* < 0.05 as significant.

## Results

The 80 cats of private owners that were analyzed in this study represented almost a quarter of the feline population in Maio Island [18]. Data collected regarding epidemiological information are shown in Table 1.

**Table 1:** Comparison of prevalence of *H. felis* in cats from Maio Island in association with different epidemiological data

Variable	No. of cats	No. of *H. felis*-positive cats (%)
Age		
Kitten (≥ 6 months to < 1 year-old)	24	6 (25.0)
Adult (≥ 1 year-old)	56	6 (10.7)
Sex		
Male	42	9 (28.1)
Female	38	3 (7.9)
Village of origin		
Morro	5	–
Calheta	20	4 (20.0)
Praia Gonçalo	3	1 (33.3)
Pedro Vaz	6	1 (16.7)
Alcatraz	5	–
Pilão Cão	1	–
Ribeira D. João	8	–
Figueira da Horta	4	–
Barreiro	10	1 (10.0)
Cidade do Porto Inglês	18	5 (27.8)
Presence of ticks		
Yes	4	2 (50.0)
No	76	10 (13.2)
Presence of fleas		
Yes	24	5 (20.8)
No	56	7 (12.5)

No circulating gamonts or morulae were detected by microscopic screening on BCS. Based on molecular analysis, 12 cats (15.0%; 95% CI: 7.2–22.8%) were positive by PCR for the presence of *Hepatozoon* spp. All 12 sequences obtained from the PCR positive cats were identical and *H. felis* was identified by BLAST analysis showing 100% nucleotide identity with *H. felis* sequences from Spanish cats available in GenBank (accession numbers AY628681 and AY620232).

**Fig. 1:**
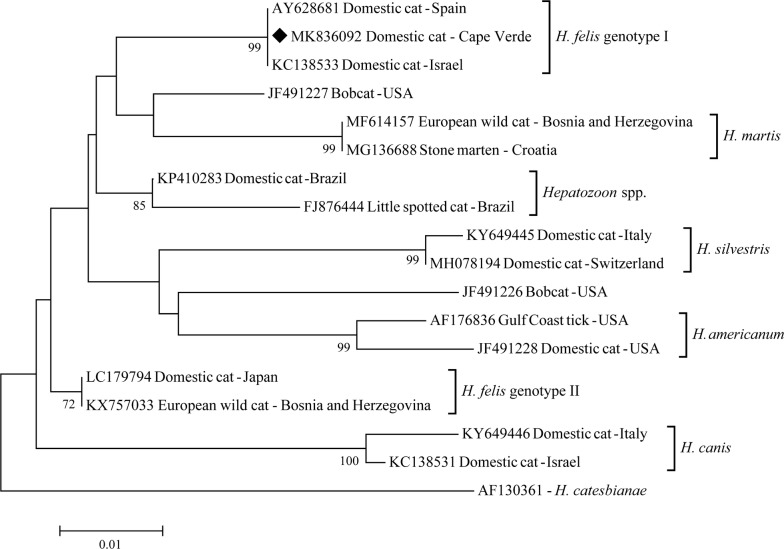
Phylogenetic tree based on 582 bp of the *18S* rRNA gene of *H. felis* in cats from Maio Island and a selection of representative *H. felis*, *H. americanum*, *H. canis*, *H. silvestris*, *H. martis* and *Hepatozoon* spp. sequences from felids retrieved from GenBank. *Hepatozoon catesbianeae* was used as the outgroup. Molecular evolutionary genetic analysis was performed with MEGA7 using the NJ method. Distances were computed using the Kimura 2-parameter model. Bootstrap values > 70% are shown. Host, country and GenBank accession number are shown for all sequences; a diamond indicates the representative *H. felis* sequence of cats from Maio Island

Phylogeny showed that *H. felis* sequence found in cats from Cape Verde clustered with *H. felis* sequences previously reported as *H. felis* genotype I, in a separate cluster from the *H. felis* genotype II sequences (Fig. 1) [12]. Similar results were obtained using the NJ and the ML methods.

Almost all (264/269) feline *Hepatozoon* sequences retrieved from GenBank and included in our comprehensive dataset clustered with previously reported *Hepatozoon* species and *H. felis* genotypes with a high phylogenetic support. The only exceptions were 5 feline *Hepatozoon* spp. sequences retrieved from domestic and wild felids in Brazil and from bobcats in the USA, that clustered separately from the *H. felis* genotypes I and II and from selected *H. canis*, *H. silvestris*, *H. americanum* and *H. martis* sequences (Fig. 1; detailed results are reported in Additional file 1: Table S1).

The geographical distribution and host species of all feline *Hepatozoon* sequences from the comprehensive dataset comprising *H. felis* genotype I and genotype II identified in the phylogenetic analysis and the representative sequence of *H. felis* genotype I from Cape Verde cats are reported in Table 2 (detailed results are reported in Additional file 1: Table S1).

**Table 2:** Geographical distribution and hosts for 248 sequences retrieved from GenBank and identified by phylogenetic analysis as *H. felis* genotype I and genotype II and the representative *Hepatozoon* sequence from Cape Verde cats

Continent	Country	Host	*H. felis* genotype I (*n*)	*H. felis* genotype II (*n*)	References^a^
Europe	Bosnia and Herzegovina	European wild cat	1	1	[40, 41]
	Croatia	Tick	1	–	MH656727
	Italy	Domestic cat	4	–	[5, 38]
	Portugal	Domestic cat	13	–	[48]
		Tick	4	–	[47]
	Spain	Domestic cat	2	–	[32]
Africa	Angola	Domestic cat	3	–	[51]
	Cape Verde	Domestic cat	1	–	This study
	Nigeria	Rat	2	–	[43]
	Zambia	Lion	2	–	[59]
		Spotted hyena	1	–	[59]
Asia	Cyprus	Domestic cat	28	–	[7]; KX808658- KX808671
	Israel	Domestic cat	5	–	[13]
		Flea	4	–	[60]
	Turkey	Tick	2	2	[26, 27, 44]
	India	Domestic cat	2	–	[12]
		Asiatic lion	1	5	[12]; KX017290
		Bengal tiger	2	–	[12]
		Indian leopard	–	2	[12]
	Thailand	Flat-headed cat	–	1	[54]
		Leopard cat	1	–	[55]
		Tick	1	–	[31]
	Republic of Korea	Leopard cat	1	2	[46]
	Japan	Domestic cat	–	6	[42]
		Amur leopard cat	–	28	[57]
		Iriomote cat	–	52	[53, 57]
		Tick	–	53	[58]
South America	Argentina	Pampas fox	1	–	[39]
		South American gray fox	1	–	[50]
		Tick	1	–	[50]
	Brazil	Domestic cat	–	2	[30, 37]
		Jaguar	–	6	[37]
		Little spotted cat	–	2	FJ876445–FJ876446
		Neotropical felid	–	2	[49]
		Ocelot	–	1	[56]

^a^GenBank accession number is provided for otherwise unpublished sequences

DNA from bacteria of the family *Anaplasmataceae* and the genus *Bartonella* was not detected in our survey. A 3.4% maximum possible prevalence in the total cat population of Maio Island of approximately 350 animals [18] was calculated for these pathogens with negative PCR results in all the samples tested.

No statistically significant differences were found between the prevalence of *H. felis* infection and the variables tested (Table 1).

## Discussion

To our knowledge, this is the first survey on feline VBPs in the Cape Verde archipelago, comprising small tropical islands with a confined and stable population of animals, with all year long presence of the vectors. Our results show that *H. felis* was present in the cat population of Maio Island.

The detection of *H. felis* by molecular techniques but not by microscopy on blood smears confirms that diagnosis of *H. felis* infection in blood samples should rely on the PCR technique. This is because microscopy of blood smears is not sensitive enough for the detection of *Hepatozoon* gamonts, due to the limited level of parasitemia, with less than 1% of neutrophils and monocytes containing gamonts [61–63]. In our study, the use of BCS, previously reported in dogs as providing an increase in sensitivity over direct blood smears in *H. canis* detection [64], did not improve diagnostic performance, further confirming that microscopy is not suitable for *Hepatozoon* diagnosis in feline blood samples.

The finding of *H. felis* as the unique species of *Hepatozoon* infecting domestic cats in Maio Island is in agreement with previous observations on *H. felis* being the most frequent *Hepatozoon* species reported in cats, especially in geographical areas with endemic infection [13]. Indeed, despite the presence of *H. canis* in dogs in Maio Island [15], only *H. felis* was found in cats from this island.

Among the variable prevalence estimations for *H. felis* that have been found in cats in different countries [5, 7, 38, 42, 61], the 15% prevalence of *H. felis* reported in Maio Island resembles that reported in domestic cats in Barcelona, Spain (16%) [65] and in Portugal (16%) [11].

The identification by phylogenetic analysis of *H. felis* genotype I in cats from Maio Island was not surprising, as this is the most frequent *H. felis* genotype reported in cats [5, 12, 13, 32, 38, 41, 48]. The presence of the *H. felis* genotype I cluster distinct from the *H. felis* genotype II cluster is in agreement with previous studies [12]. The two strongly supported distinct clades for *H. felis* genoype I and genotype II also suggest that they probably represent two different *Hepatozoon* species. Therefore, only one *H. felis* genotype may be referred to as *H. felis* while the other may be indicative of a different *Hepatozoon* species, as recently proposed [5].

Despite the wide geographical distribution of both *H. felis* genotypes, geographical differences between the *H. felis* genotype I and II were observed, as recently suggested [30]. Analysis on feline *Hepatozoon* sequences retrieved from GenBank showed that *H. felis* genotype I was the only genotype identified in Africa and the Middle East and was also the most frequent genotype observed in the Mediterranean basin. On the other hand, *H. felis* genotype II was the only *H. felis* genotype found in Brazil and Japan, as previously reported [37, 42, 49, 56–58].

Our analysis assessed the absence of host specificity for feline *Hepatozoon* species, confirming that these parasites are not considered to be specific of the suborders (felids and canids) of Carnivora [13, 59]. Indeed, both genotypes of *H. felis* were previously reported in domestic and wild felids and *H. felis* genotype I was also reported in other mammals (foxes, rodents, hyenas) [39, 43, 50, 59]. Our phylogenetic study also showed that felids can be infected by *Hepatozoon* species typically observed in dogs (*H. canis*, *H. americanum*) or in martens (*H. martis*), as previously reported [28, 33, 61, 66]. Moreover, domestic cats can also be infected by *H. silvestris* previously reported only in wild felids [38, 40, 41, 45]. However, in Maio Island only *H. felis* was detected in our study. Further studies are needed to understand the differences between the *H. felis* genotypes and more generally the *Hepatozoon* spp. infecting felids in terms of their etiological and epidemiological significance.

A few risk factors have been identified to date for feline hepatozoonosis [61]. The previously reported association between infection and outdoor access, suggesting transmission by hematophagous arthropods or predation as demonstrated for other *Hepatozoon* spp. [13], could not be confirmed in our study because all privately-owned cats from Maio Island had a mixed indoor–outdoor lifestyle. Despite the fact that *Rh. sanguineus* (*s.l.*) is the only hard tick present in the Cape Verde archipelago [16] and has been suggested as candidate vector of *H. felis* infection, resembling the well-established scenario of *H. canis* in dogs [47, 67], the association of *H. felis* infection in cats with presence of ticks was not found in our investigation. Further studies on cats and vectors are needed to confirm the route of transmission of *H. felis* infection in Maio Island. Our results showed that neither age nor sex was associated with *H. felis* infection in cats, confirming previous results [13]. The absence of a significant association between *H. felis* infection in cats and location suggests that the infection was distributed on the whole island, probably reflecting animal movement or the wide distribution of ticks [16]. The presence of *H. felis* in apparently healthy cats from Maio Island was not surprising, since *H. felis* is commonly associated with subclinical symptoms [13].

Regarding the negative PCR results for the other VBPs tested in this study, it has to be taken into account that even if almost one quarter of the whole population of cats in Maio Island was studied, this sampling size is not adequate for drawing any definitive conclusion on absence or very low prevalence of these VBPs in the cat population.

Despite the fact that *Ehrlichia canis* and *Anaplasma platys* were previously reported in dogs from Maio Island, the negative PCR results in cats supports findings indicating that cats are less frequently affected by arthropod-borne pathogens than dogs, likely due to the lower rate of tick infestation generally reported in cats compared to dogs [2]. Indeed, in our study ticks were detected in only four (5%) cats. The negative PCR result of *Anaplasma phagocytophilum* in cats is also in agreement with negative results in dogs of the same areas, as the vector *Ixodes ricinus* has not been reported in the archipelago [15, 16].

The negative PCR results for *Bartonella* spp. were not expected, since feline bartonellosis has been reported in cats worldwide and transmission in cats can occur by the cat flea (*Ctenocephalides felis*) [68]. Despite the fact that fleas were observed in 24 (30%) cats from Maio Island, the calculated 3.4% maximum possible prevalence of feline bartonellosis is in agreement with low prevalence rates of 0.3, 0.7, 0.9 and 2.9% that have been reported in Spain, Albania, Algeria and Portugal, respectively [8, 48, 69, 70].

## Conclusions

To our knowledge, this is the first time that infection with *H. felis* has been reported in the Cape Verde archipelago in cats; *H. felis* genotype I was observed, reinforcing previous findings that, to date, only this genotype has been reported in Africa. The presence of *H. felis* in Maio Island should encourage a campaign of VBPs monitoring in the Cape Verde archipelago, with special emphasis on the investigation in animals and vectors, possible routes of infection and impact of infection in cats, especially to obtain a wider perspective on feline hepatozoonosis.

## Additional file

**Additional file 1: Table S1.** Data on feline *Hepatozoon* sequences: *Hepatozoon* species classification based on results of phylogenetic analysis of 269 selected *H. felis* sequences from mammal species and vectors, along with *H. canis*, *H. silvestris* and *Hepatozoon* spp. sequences from felids retrieved from GenBank and the representative *Hepatozoon* spp. sequence from Cape Verde cats.

## Data Availability

All datasets supporting the conclusions of this article are included within the article and its additional file. Raw data are available from the corresponding author upon request. A representative sequence was submitted to the GenBank database under the accession number MK836092.

## Abbreviations

PCR: polymerase chain reaction
VBDs: vector-borne diseases
BCS: buffy coat smear
NJ: neighbor-joining
ML: maximum likelihood
