# Supplementary material for: Molecular detection of Hepatozoon felis in cats from Maio Island, Republic of Cape Verde and global distribution of feline hepatozoonosis
Source: Parasit Vectors. 2019 Jun 11;12:294. doi: 10.1186/s13071-019-3551-3 (PMC6560739; doi:10.1186/s13071-019-3551-3)
Supplement: Supplementary file 1 — Additional file 1: Table S1. Data on feline Hepatozoon sequences: Hepatozoon species classification based on results of phylogenetic analysis of 269 selected H. felis sequences from mammal species and vectors, along with H. canis, H. silvestris and Hepatozoon spp. sequences from felids retrieved from GenBank and the representative Hepatozoon spp. sequence from Cape Verde cats. [file 13071_2019_3551_MOESM1_ESM.docx]

**Additional file 1: Table S1.** Data on feline *Hepatozoon* sequences: *Hepatozoon* species classification based on results of phylogenetic analysis of 269 selected *H. felis* sequences from mammal species and vectors, along with *H. canis*, *H. silvestris* and *Hepatozoon* spp. sequences from felids retrieved from GenBank and the representative *Hepatozoon* spp. sequence from Cape Verde cats.

| **Region** | **Country** | **Host** | **Accession number** | **Reference^a^** | ***Hepatozoon* species by phylogeny** |
| --- | --- | --- | --- | --- | --- |
| Eastern Africa | Zambia | Lion (*Panthera leo*) | KF270665 | 59 | *H. felis* genotype I |
| Eastern Africa | Zambia | Lion (*Panthera leo*) | KF270668 | 59 | *H. felis* genotype I |
| Eastern Africa | Zambia | Spotted hyena (*Crocuta crocuta*) | KF270660 | 59 | *H. felis* genotype I |
| Middle Africa | Angola | Domestic cat (*Felis catus domesticus*) | MG386482 | 51 | *H. felis* genotype I |
| Middle Africa | Angola | Domestic cat (*Felis catus domesticus*) | MG386483 | 51 | *H. felis* genotype I |
| Middle Africa | Angola | Domestic cat (*Felis catus domesticus*) | MG386484 | 51 | *H. felis* genotype I |
| Western Africa | Cape Verde | Domestic cat (*Felis catus domesticus*) | MK836092 | This study | *H. felis* genotype I |
| Western Africa | Nigeria | Rat (*Rattus norvegicus*) | MG786593 | 43 | *H. felis* genotype I |
| Western Africa | Nigeria | Rat (*Rattus rattus*) | MG786594 | 43 | *H. felis* genotype I |
| Northern America | USA | Bobcat (*Lynx rufus*) | JF491226 | 28 | *Hepatozoon* spp.^b^ |
| Northern America | USA | Bobcat (*Lynx rufus*) | JF491227 | 28 | *Hepatozoon* spp.^c^ |
| Northern America | USA | Domestic cat (*Felis catus domesticus*) | JF491228 | 28 | *H. americanum* |
| South America | Argentina | Pampas fox (*Lycalopex gymnocercus*) | HQ020489 | 39 | *H. felis* genotype I |
| South America | Argentina | South American gray fox (*Lycalopex griseus*) | MK049948 | 50 | *H. felis* genotype I |
| South America | Argentina | Tick (*Amblyomma tigrinum*, from fox) | MK049950 | 50 | *H. felis* genotype I |
| South America | Brazil | Domestic cat (*Felis catus domesticus*) | DQ315565 | 52 | *H. canis* |
| South America | Brazil | Domestic cat (*Felis catus domesticus*) | DQ315566 | 52 | *H. canis* |
| South America | Brazil | Domestic cat (*Felis catus domesticus*) | KP410283 | 29 | *Hepatozoon* spp.^d^ |
| South America | Brazil | Domestic cat (*Felis catus domesticus*) | KM435071 | 30 | *H. felis* genotype II |
| South America | Brazil | Domestic cat (*Felis catus domesticus*) | JN123435 | 34 | *Hepatozoon* spp. ^d^ |
| South America | Brazil | Domestic cat (*Felis catus domesticus*) | KU232306 | 37 | *H. felis* genotype II |
| South America | Brazil | Jaguar (*Panthera onca*) | KU232302 | 37 | *H. felis* genotype II |
| South America | Brazil | Jaguar (*Panthera onca*) | KU232303 | 37 | *H. felis* genotype II |
| South America | Brazil | Jaguar (*Panthera onca*) | KU232304 | 37 | *H. felis* genotype II |
| South America | Brazil | Jaguar (*Panthera onca*) | KU232305 | 37 | *H. felis* genotype II |
| South America | Brazil | Jaguar (*Panthera onca*) | KU232307 | 37 | *H. felis* genotype II |
| South America | Brazil | Jaguar (*Panthera onca*) | KU232308 | 37 | *H. felis* genotype II |
| South America | Brazil | Little spotted cat (*Leopardus tigrinus*) | FJ876445 | unpublished | *H. felis* genotype II |
| South America | Brazil | Little spotted cat (*Leopardus tigrinus*) | FJ876446 | unpublished | *H. felis* genotype II |
| South America | Brazil | Little spotted cat (*Leopardus tigrinus*) | FJ876444 | unpublished | *Hepatozoon* spp. ^d^ |
| South America | Brazil | Neotropical felid | EU028344 | 49 | *H. felis* genotype II |
| South America | Brazil | Neotropical felid | EU267606 | 49 | *H. felis* genotype II |
| South America | Brazil | Ocelot (*Leopardus pardalis*) | KY684005 | 56 | *H. felis* genotype II |
| Eastern Asia | Japan | Domestic cat (*Felis catus domesticus*) | LC179794 | 42 | *H. felis* genotype II |
| Eastern Asia | Japan | Domestic cat (*Felis catus domesticus*) | LC179795 | 42 | *H. felis* genotype II |
| Eastern Asia | Japan | Domestic cat (*Felis catus domesticus*) | LC179796 | 42 | *H. felis* genotype II |
| Eastern Asia | Japan | Domestic cat (*Felis catus domesticus*) | LC179797 | 42 | *H. felis* genotype II |
| Eastern Asia | Japan | Domestic cat (*Felis catus domesticus*) | LC179798 | 42 | *H. felis* genotype II |
| Eastern Asia | Japan | Domestic cat (*Felis catus domesticus*) | LC179799 | 42 | *H. felis* genotype II |
| Eastern Asia | Japan | Iriomote cat (*Prionailurus bengalensis iriomotensis*) | AB636285 | 53 | *H. felis* genotype II |
| Eastern Asia | Japan | Iriomote cat (*Prionailurus bengalensis iriomotensis*) | AB636286 | 53 | *H. felis* genotype II |
| Eastern Asia | Japan | Iriomote cat (*Prionailurus bengalensis iriomotensis*) | AB636287 | 53 | *H. felis* genotype II |
| Eastern Asia | Japan | Iriomote cat (*Prionailurus bengalensis iriomotensis*) | AB771501 | 57 | *H. felis* genotype II |
| Eastern Asia | Japan | Iriomote cat (*Prionailurus bengalensis iriomotensis*) | AB771502 | 57 | *H. felis* genotype II |
| Eastern Asia | Japan | Iriomote cat (*Prionailurus bengalensis iriomotensis*) | AB771503 | 57 | *H. felis* genotype II |
| Eastern Asia | Japan | Iriomote cat (*Prionailurus bengalensis iriomotensis*) | AB771504 | 57 | *H. felis* genotype II |
| Eastern Asia | Japan | Iriomote cat (*Prionailurus bengalensis iriomotensis*) | AB771505 | 57 | *H. felis* genotype II |
| Eastern Asia | Japan | Iriomote cat (*Prionailurus bengalensis iriomotensis*) | AB771506 | 57 | *H. felis* genotype II |
| Eastern Asia | Japan | Iriomote cat (*Prionailurus bengalensis iriomotensis*) | AB771507 | 57 | *H. felis* genotype II |
| Eastern Asia | Japan | Iriomote cat (*Prionailurus bengalensis iriomotensis*) | AB771508 | 57 | *H. felis* genotype II |
| Eastern Asia | Japan | Iriomote cat (*Prionailurus bengalensis iriomotensis*) | AB771509 | 57 | *H. felis* genotype II |
| Eastern Asia | Japan | Iriomote cat (*Prionailurus bengalensis iriomotensis*) | AB771510 | 57 | *H. felis* genotype II |
| Eastern Asia | Japan | Iriomote cat (*Prionailurus bengalensis iriomotensis*) | AB771511 | 57 | *H. felis* genotype II |
| Eastern Asia | Japan | Iriomote cat (*Prionailurus bengalensis iriomotensis*) | AB771512 | 57 | *H. felis* genotype II |
| Eastern Asia | Japan | Iriomote cat (*Prionailurus bengalensis iriomotensis*) | AB771513 | 57 | *H. felis* genotype II |
| Eastern Asia | Japan | Iriomote cat (*Prionailurus bengalensis iriomotensis*) | AB771514 | 57 | *H. felis* genotype II |
| Eastern Asia | Japan | Iriomote cat (*Prionailurus bengalensis iriomotensis*) | AB771515 | 57 | *H. felis* genotype II |
| Eastern Asia | Japan | Iriomote cat (*Prionailurus bengalensis iriomotensis*) | AB771516 | 57 | *H. felis* genotype II |
| Eastern Asia | Japan | Iriomote cat (*Prionailurus bengalensis iriomotensis*) | AB771517 | 57 | *H. felis* genotype II |
| Eastern Asia | Japan | Iriomote cat (*Prionailurus bengalensis iriomotensis*) | AB771518 | 57 | *H. felis* genotype II |
| Eastern Asia | Japan | Iriomote cat (*Prionailurus bengalensis iriomotensis*) | AB771519 | 57 | *H. felis* genotype II |
| Eastern Asia | Japan | Iriomote cat (*Prionailurus bengalensis iriomotensis*) | AB771520 | 57 | *H. felis* genotype II |
| Eastern Asia | Japan | Iriomote cat (*Prionailurus bengalensis iriomotensis*) | AB771521 | 57 | *H. felis* genotype II |
| Eastern Asia | Japan | Iriomote cat (*Prionailurus bengalensis iriomotensis*) | AB771522 | 57 | *H. felis* genotype II |
| Eastern Asia | Japan | Iriomote cat (*Prionailurus bengalensis iriomotensis*) | AB771523 | 57 | *H. felis* genotype II |
| Eastern Asia | Japan | Iriomote cat (*Prionailurus bengalensis iriomotensis*) | AB771524 | 57 | *H. felis* genotype II |
| Eastern Asia | Japan | Iriomote cat (*Prionailurus bengalensis iriomotensis*) | AB771525 | 57 | *H. felis* genotype II |
| Eastern Asia | Japan | Iriomote cat (*Prionailurus bengalensis iriomotensis*) | AB771526 | 57 | *H. felis* genotype II |
| Eastern Asia | Japan | Iriomote cat (*Prionailurus bengalensis iriomotensis*) | AB771527 | 57 | *H. felis* genotype II |
| Eastern Asia | Japan | Iriomote cat (*Prionailurus bengalensis iriomotensis*) | AB771528 | 57 | *H. felis* genotype II |
| Eastern Asia | Japan | Iriomote cat (*Prionailurus bengalensis iriomotensis*) | AB771529 | 57 | *H. felis* genotype II |
| Eastern Asia | Japan | Iriomote cat (*Prionailurus bengalensis iriomotensis*) | AB771530 | 57 | *H. felis* genotype II |
| Eastern Asia | Japan | Iriomote cat (*Prionailurus bengalensis iriomotensis*) | AB771531 | 57 | *H. felis* genotype II |
| Eastern Asia | Japan | Iriomote cat (*Prionailurus bengalensis iriomotensis*) | AB771532 | 57 | *H. felis* genotype II |
| Eastern Asia | Japan | Iriomote cat (*Prionailurus bengalensis iriomotensis*) | AB771533 | 57 | *H. felis* genotype II |
| Eastern Asia | Japan | Iriomote cat (*Prionailurus bengalensis iriomotensis*) | AB771534 | 57 | *H. felis* genotype II |
| Eastern Asia | Japan | Iriomote cat (*Prionailurus bengalensis iriomotensis*) | AB771535 | 57 | *H. felis* genotype II |
| Eastern Asia | Japan | Iriomote cat (*Prionailurus bengalensis iriomotensis*) | AB771536 | 57 | *H. felis* genotype II |
| Eastern Asia | Japan | Iriomote cat (*Prionailurus bengalensis iriomotensis*) | AB771537 | 57 | *H. felis* genotype II |
| Eastern Asia | Japan | Iriomote cat (*Prionailurus bengalensis iriomotensis*) | AB771538 | 57 | *H. felis* genotype II |
| Eastern Asia | Japan | Iriomote cat (*Prionailurus bengalensis iriomotensis*) | AB771539 | 57 | *H. felis* genotype II |
| Eastern Asia | Japan | Iriomote cat (*Prionailurus bengalensis iriomotensis*) | AB771540 | 57 | *H. felis* genotype II |
| Eastern Asia | Japan | Iriomote cat (*Prionailurus bengalensis iriomotensis*) | AB771541 | 57 | *H. felis* genotype II |
| Eastern Asia | Japan | Iriomote cat (*Prionailurus bengalensis iriomotensis*) | AB771542 | 57 | *H. felis* genotype II |
| Eastern Asia | Japan | Iriomote cat (*Prionailurus bengalensis iriomotensis*) | AB771543 | 57 | *H. felis* genotype II |
| Eastern Asia | Japan | Iriomote cat (*Prionailurus bengalensis iriomotensis*) | AB771544 | 57 | *H. felis* genotype II |
| Eastern Asia | Japan | Iriomote cat (*Prionailurus bengalensis iriomotensis*) | AB771545 | 57 | *H. felis* genotype II |
| Eastern Asia | Japan | Iriomote cat (*Prionailurus bengalensis iriomotensis*) | AB771546 | 57 | *H. felis* genotype II |
| Eastern Asia | Japan | Iriomote cat (*Prionailurus bengalensis iriomotensis*) | AB771547 | 57 | *H. felis* genotype II |
| Eastern Asia | Japan | Iriomote cat (*Prionailurus bengalensis iriomotensis*) | AB771548 | 57 | *H. felis* genotype II |
| Eastern Asia | Japan | Iriomote cat (*Prionailurus bengalensis iriomotensis*) | AB771549 | 57 | *H. felis* genotype II |
| Eastern Asia | Japan | Amur leopard cat (*Prionailurus bengalensis euptilurus*) | AB771550 | 57 | *H. felis* genotype II |
| Eastern Asia | Japan | Amur leopard cat (*Prionailurus bengalensis euptilurus*) | AB771551 | 57 | *H. felis* genotype II |
| Eastern Asia | Japan | Amur leopard cat (*Prionailurus bengalensis euptilurus*) | AB771552 | 57 | *H. felis* genotype II |
| Eastern Asia | Japan | Amur leopard cat (*Prionailurus bengalensis euptilurus*) | AB771553 | 57 | *H. felis* genotype II |
| Eastern Asia | Japan | Amur leopard cat (*Prionailurus bengalensis euptilurus*) | AB771554 | 57 | *H. felis* genotype II |
| Eastern Asia | Japan | Amur leopard cat (*Prionailurus bengalensis euptilurus*) | AB771555 | 57 | *H. felis* genotype II |
| Eastern Asia | Japan | Amur leopard cat (*Prionailurus bengalensis euptilurus*) | AB771556 | 57 | *H. felis* genotype II |
| Eastern Asia | Japan | Amur leopard cat (*Prionailurus bengalensis euptilurus*) | AB771557 | 57 | *H. felis* genotype II |
| Eastern Asia | Japan | Amur leopard cat (*Prionailurus bengalensis euptilurus*) | AB771558 | 57 | *H. felis* genotype II |
| Eastern Asia | Japan | Amur leopard cat (*Prionailurus bengalensis euptilurus*) | AB771559 | 57 | *H. felis* genotype II |
| Eastern Asia | Japan | Amur leopard cat (*Prionailurus bengalensis euptilurus*) | AB771560 | 57 | *H. felis* genotype II |
| Eastern Asia | Japan | Amur leopard cat (*Prionailurus bengalensis euptilurus*) | AB771561 | 57 | *H. felis* genotype II |
| Eastern Asia | Japan | Amur leopard cat (*Prionailurus bengalensis euptilurus*) | AB771562 | 57 | *H. felis* genotype II |
| Eastern Asia | Japan | Amur leopard cat (*Prionailurus bengalensis euptilurus*) | AB771563 | 57 | *H. felis* genotype II |
| Eastern Asia | Japan | Amur leopard cat (*Prionailurus bengalensis euptilurus*) | AB771564 | 57 | *H. felis* genotype II |
| Eastern Asia | Japan | Amur leopard cat (*Prionailurus bengalensis euptilurus*) | AB771565 | 57 | *H. felis* genotype II |
| Eastern Asia | Japan | Amur leopard cat (*Prionailurus bengalensis euptilurus*) | AB771566 | 57 | *H. felis* genotype II |
| Eastern Asia | Japan | Amur leopard cat (*Prionailurus bengalensis euptilurus*) | AB771567 | 57 | *H. felis* genotype II |
| Eastern Asia | Japan | Amur leopard cat (*Prionailurus bengalensis euptilurus*) | AB771568 | 57 | *H. felis* genotype II |
| Eastern Asia | Japan | Amur leopard cat (*Prionailurus bengalensis euptilurus*) | AB771569 | 57 | *H. felis* genotype II |
| Eastern Asia | Japan | Amur leopard cat (*Prionailurus bengalensis euptilurus*) | AB771570 | 57 | *H. felis* genotype II |
| Eastern Asia | Japan | Amur leopard cat (*Prionailurus bengalensis euptilurus*) | AB771571 | 57 | *H. felis* genotype II |
| Eastern Asia | Japan | Amur leopard cat (*Prionailurus bengalensis euptilurus*) | AB771572 | 57 | *H. felis* genotype II |
| Eastern Asia | Japan | Amur leopard cat (*Prionailurus bengalensis euptilurus*) | AB771573 | 57 | *H. felis* genotype II |
| Eastern Asia | Japan | Amur leopard cat (*Prionailurus bengalensis euptilurus*) | AB771574 | 57 | *H. felis* genotype II |
| Eastern Asia | Japan | Amur leopard cat (*Prionailurus bengalensis euptilurus*) | AB771575 | 57 | *H. felis* genotype II |
| Eastern Asia | Japan | Amur leopard cat (*Prionailurus bengalensis euptilurus*) | AB771576 | 57 | *H. felis* genotype II |
| Eastern Asia | Japan | Amur leopard cat (*Prionailurus bengalensis euptilurus*) | AB771577 | 57 | *H. felis* genotype II |
| Eastern Asia | Japan | Tick (*Haemaphysalis longicornis*, from Iriomote cat) | AB983385 | 58 | *H. felis* genotype II |
| Eastern Asia | Japan | Tick (*Haemaphysalis hystricis*, from Iriomote cat) | AB983386 | 58 | *H. felis* genotype II |
| Eastern Asia | Japan | Tick (*Haemaphysalis longicornis*, from Iriomote cat) | AB983387 | 58 | *H. felis* genotype II |
| Eastern Asia | Japan | Tick (*Haemaphysalis hystricis*, from Iriomote cat) | AB983388 | 58 | *H. felis* genotype II |
| Eastern Asia | Japan | Tick (*Haemaphysalis hystricis* from Iriomote cat) | AB983389 | 58 | *H. felis* genotype II |
| Eastern Asia | Japan | Tick (*Haemaphysalis hystricis,* from Iriomote cat) | AB983390 | 58 | *H. felis* genotype II |
| Eastern Asia | Japan | Tick (*Haemaphysalis hystricis*, from Iriomote cat) | AB983391 | 58 | *H. felis* genotype II |
| Eastern Asia | Japan | Tick (*Amblyomma testudinarium*, from Iriomote cat) | AB983392 | 58 | *H. felis* genotype II |
| Eastern Asia | Japan | Tick (*Haemaphysalis hystricis*, from Iriomote cat) | AB983393 | 58 | *H. felis* genotype II |
| Eastern Asia | Japan | Tick (*Haemaphysalis hystricis*, from Iriomote cat) | AB983394 | 58 | *H. felis* genotype II |
| Eastern Asia | Japan | Tick (*Amblyomma testudinarium,* from Iriomote cat) | AB983395 | 58 | *H. felis* genotype II |
| Eastern Asia | Japan | Tick (*Haemaphysalis hystricis*, from Iriomote cat) | AB983396 | 58 | *H. felis* genotype II |
| Eastern Asia | Japan | Tick (*Haemaphysalis hystricis*, from Iriomote cat) | AB983397 | 58 | *H. felis* genotype II |
| Eastern Asia | Japan | Tick (*Haemaphysalis hystricis*, from Iriomote cat) | AB983398 | 58 | *H. felis* genotype II |
| Eastern Asia | Japan | Tick (*Haemaphysalis hystricis*, from Iriomote cat) | AB983399 | 58 | *H. felis* genotype II |
| Eastern Asia | Japan | Tick (*Haemaphysalis hystricis*, from Iriomote cat) | AB983400 | 58 | *H. felis* genotype II |
| Eastern Asia | Japan | Tick (*Haemaphysalis hystricis*, from Iriomote cat) | AB983401 | 58 | *H. felis* genotype II |
| Eastern Asia | Japan | Tick (*Haemaphysalis hystricis*, from Iriomote cat) | AB983402 | 58 | *H. felis* genotype II |
| Eastern Asia | Japan | Tick (*Amblyomma testudinarium,* from Iriomote cat) | AB983403 | 58 | *H. felis* genotype II |
| Eastern Asia | Japan | Tick (*Haemaphysalis longicornis*, from Iriomote cat) | AB983404 | 58 | *H. felis* genotype II |
| Eastern Asia | Japan | Tick (*Haemaphysalis hystricis*, from Iriomote cat) | AB983405 | 58 | *H. felis* genotype II |
| Eastern Asia | Japan | Tick (*Haemaphysalis hystricis*, from Iriomote cat) | AB983406 | 58 | *H. felis* genotype II |
| Eastern Asia | Japan | Tick (*Haemaphysalis hystricis*, from Iriomote cat) | AB983407 | 58 | *H. felis* genotype II |
| Eastern Asia | Japan | Tick (*Ixodes tanuki*, from Amur leopard cat) | AB983408 | 58 | *H. felis* genotype II |
| Eastern Asia | Japan | Tick (*Ixodes tanuki*, from Amur leopard cat) | AB983409 | 58 | *H. felis* genotype II |
| Eastern Asia | Japan | Tick (*Haemaphysalis megaspinosa*, from Amur leopard cat) | AB983410 | 58 | *H. felis* genotype II |
| Eastern Asia | Japan | Tick (*Haemaphysalis campanulata*, from Amur leopard cat) | AB983411 | 58 | *H. felis* genotype II |
| Eastern Asia | Japan | Tick (*Ixodes tanuki*, from Amur leopard cat) | AB983412 | 58 | *H. felis* genotype II |
| Eastern Asia | Japan | Tick (*Ixodes tanuki*, from Amur leopard cat) | AB983413 | 58 | *H. felis* genotype II |
| Eastern Asia | Japan | Tick (*Ixodes tanuki*, from Amur leopard cat) | AB983414 | 58 | *H. felis* genotype II |
| Eastern Asia | Japan | Tick (*Amblyomma testudinarium*, from Amur leopard cat) | AB983415 | 58 | *H. felis* genotype II |
| Eastern Asia | Japan | Tick (*Haemaphysalis megaspinosa*, from Amur leopard cat) | AB983416 | 58 | *H. felis* genotype II |
| Eastern Asia | Japan | Tick (*Ixodes tanuki*, from Amur leopard cat) | AB983417 | 58 | *H. felis* genotype II |
| Eastern Asia | Japan | Tick (*Ixodes tanuki*, from Amur leopard cat) | AB983418 | 58 | *H. felis* genotype II |
| Eastern Asia | Japan | Tick (*Ixodes tanuki*, from Amur leopard cat) | AB983419 | 58 | *H. felis* genotype II |
| Eastern Asia | Japan | Tick (*Ixodes tanuki*, from Amur leopard cat) | AB983420 | 58 | *H. felis* genotype II |
| Eastern Asia | Japan | Tick (*Haemaphysalis campanulata*, from Amur leopard cat) | AB983421 | 58 | *H. felis* genotype II |
| Eastern Asia | Japan | Tick (*Haemaphysalis megaspinosa*, from Amur leopard cat) | AB983422 | 58 | *H. felis* genotype II |
| Eastern Asia | Japan | Tick (*Ixodes tanuki*, from Amur leopard cat) | AB983423 | 58 | *H. felis* genotype II |
| Eastern Asia | Japan | Tick (*Ixodes tanuki*, from Amur leopard cat) | AB983424 | 58 | *H. felis* genotype II |
| Eastern Asia | Japan | Tick (*Haemaphysalis megaspinosa*, from Amur leopard cat) | AB983425 | 58 | *H. felis* genotype II |
| Eastern Asia | Japan | Tick (*Ixodes tanuki*, from Amur leopard cat) | AB983426 | 58 | *H. felis* genotype II |
| Eastern Asia | Japan | Tick (*Ixodes tanuki*, from Amur leopard cat) | AB983427 | 58 | *H. felis* genotype II |
| Eastern Asia | Japan | Tick (*Haemaphysalis megaspinosa*, from Amur leopard cat) | AB983428 | 58 | *H. felis* genotype II |
| Eastern Asia | Japan | Tick (*Ixodes tanuki*, from Amur leopard cat) | AB983429 | 58 | *H. felis* genotype II |
| Eastern Asia | Japan | Tick (*Ixodes tanuki*, from Amur leopard cat) | AB983430 | 58 | *H. felis* genotype II |
| Eastern Asia | Japan | Tick (*Ixodes tanuki*, from Amur leopard cat) | AB983431 | 58 | *H. felis* genotype II |
| Eastern Asia | Japan | Tick (*Ixodes tanuki*, from Amur leopard cat)) | AB983432 | 58 | *H. felis* genotype II |
| Eastern Asia | Japan | Tick (*Haemaphysalis megaspinosa*, from Amur leopard cat) | AB983433 | 58 | *H. felis* genotype II |
| Eastern Asia | Japan | Tick (*Haemaphysalis megaspinosa*, from Amur leopard cat) | AB983434 | 58 | *H. felis* genotype II |
| Eastern Asia | Japan | Tick (*Amblyomma testudinarium*, from Amur leopard cat) | AB983435 | 58 | *H. felis* genotype II |
| Eastern Asia | Japan | Tick (*Ixodes tanuki*, from Amur leopard cat) | AB983436 | 58 | *H. felis* genotype II |
| Eastern Asia | Japan | Tick (*Ixodes tanuki*, from Amur leopard cat) | AB983437 | 58 | *H. felis* genotype II |
| Eastern Asia | Republic of Korea | Leopard cat (*Prionailurus bengalensis*) | GQ377216 | 46 | *H. felis* genotype I |
| Eastern Asia | Republic of Korea | Leopard cat (*Prionailurus bengalensis*) | GQ377217 | 46 | *H. felis* genotype II |
| Eastern Asia | Republic of Korea | Leopard cat (*Prionailurus bengalensis*) | GQ377218 | 46 | *H. felis* genotype II |
| South-eastern Asia | Thailand | Flat-headed cat (*Prionailurus planiceps*) | GQ926901 | 54 | *H. felis* genotype II |
| South-eastern Asia | Thailand | Leopard cat (*Prionailurus bengalensis*) | GQ926902 | 55 | *H. felis* genotype I |
| South-eastern Asia | Thailand | Tick (*Rhipicephalus sanguineus,* from Asiatic lion) | KY056823 | 31 | *H. felis* genotype I |
| Southern Asia | India | Domestic cat (*Felis catus domesticus*) | JN584475 | 12 | *H. felis* genotype I |
| Southern Asia | India | Domestic cat (*Felis catus domesticus*) | JN584476 | 12 | *H. felis* genotype I |
| Southern Asia | India | Asiatic Lion (*Panthera leo leo*) | HQ829438 | 12 | *H. felis* genotype II |
| Southern Asia | India | Asiatic Lion (*Panthera leo leo*) | HQ829439 | 12 | *H. felis* genotype II |
| Southern Asia | India | Asiatic Lion (*Panthera leo leo*) | HQ829440 | 12 | *H. felis* genotype II |
| Southern Asia | India | Asiatic Lion (*Panthera leo leo*) | HQ829441 | 12 | *H. felis* genotype II |
| Southern Asia | India | Asiatic Lion (*Panthera leo leo*) | HQ829442 | 12 | *H. felis* genotype II |
| Southern Asia | India | Indian leopard (*Panthera pardus fusca*) | HQ829443 | 12 | *H. felis* genotype II |
| Southern Asia | India | Indian leopard (*Panthera pardus fusca*) | HQ829444 | 12 | *H. felis* genotype II |
| Southern Asia | India | Bengal tiger (*Panthera tigris tigris*) | HQ829445 | 12 | *H. felis* genotype I |
| Southern Asia | India | Bengal tiger (*Panthera tigris tigris*) | HQ829446 | 12 | *H. felis* genotype I |
| Southern Asia | India | Asiatic Lion (*Panthera leo leo*) | KX017290 | unpublished | *H. felis* genotype I |
| Western Asia | Cyprus | Domestic cat (*Felis catus domesticus*) | KX808658 | unpublished | *H. felis* genotype I |
| Western Asia | Cyprus | Domestic cat (*Felis catus domesticus*) | KX808659 | unpublished | *H. felis* genotype I |
| Western Asia | Cyprus | Domestic cat (*Felis catus domesticus*) | KX808660 | unpublished | *H. felis* genotype I |
| Western Asia | Cyprus | Domestic cat (*Felis catus domesticus*) | KX808661 | unpublished | *H. felis* genotype I |
| Western Asia | Cyprus | Domestic cat (*Felis catus domesticus*) | KX808662 | unpublished | *H. felis* genotype I |
| Western Asia | Cyprus | Domestic cat (*Felis catus domesticus*) | KX808663 | unpublished | *H. felis* genotype I |
| Western Asia | Cyprus | Domestic cat (*Felis catus domesticus*) | KX808664 | unpublished | *H. felis* genotype I |
| Western Asia | Cyprus | Domestic cat (*Felis catus domesticus*) | KX808665 | unpublished | *H. felis* genotype I |
| Western Asia | Cyprus | Domestic cat (*Felis catus domesticus*) | KX808666 | unpublished | *H. felis* genotype I |
| Western Asia | Cyprus | Domestic cat (*Felis catus domesticus*) | KX808667 | unpublished | *H. felis* genotype I |
| Western Asia | Cyprus | Domestic cat (*Felis catus domesticus*) | KX808668 | unpublished | *H. felis* genotype I |
| Western Asia | Cyprus | Domestic cat (*Felis catus domesticus*) | KX808669 | unpublished | *H. felis* genotype I |
| Western Asia | Cyprus | Domestic cat (*Felis catus domesticus*) | KX808670 | unpublished | *H. felis* genotype I |
| Western Asia | Cyprus | Domestic cat (*Felis catus domesticus*) | KX808671 | unpublished | *H. felis* genotype I |
| Western Asia | Cyprus | Domestic cat (*Felis catus domesticus*) | KY215805 | 7 | *H. felis* genotype I |
| Western Asia | Cyprus | Domestic cat (*Felis catus domesticus*) | KY215806 | 7 | *H. felis* genotype I |
| Western Asia | Cyprus | Domestic cat (*Felis catus domesticus*) | KY215807 | 7 | *H. felis* genotype I |
| Western Asia | Cyprus | Domestic cat (*Felis catus domesticus*) | KY215808 | 7 | *H. felis* genotype I |
| Western Asia | Cyprus | Domestic cat (*Felis catus domesticus*) | KY215809 | 7 | *H. felis* genotype I |
| Western Asia | Cyprus | Domestic cat (*Felis catus domesticus*) | KY215810 | 7 | *H. felis* genotype I |
| Western Asia | Cyprus | Domestic cat (*Felis catus domesticus*) | KY215811 | 7 | *H. felis* genotype I |
| Western Asia | Cyprus | Domestic cat (*Felis catus domesticus*) | KY215812 | 7 | *H. felis* genotype I |
| Western Asia | Cyprus | Domestic cat (*Felis catus domesticus*) | KY215813 | 7 | *H. felis* genotype I |
| Western Asia | Cyprus | Domestic cat (*Felis catus domesticus*) | KY215814 | 7 | *H. felis* genotype I |
| Western Asia | Cyprus | Domestic cat (*Felis catus domesticus*) | KY215815 | 7 | *H. felis* genotype I |
| Western Asia | Cyprus | Domestic cat (*Felis catus domesticus*) | KY215816 | 7 | *H. felis* genotype I |
| Western Asia | Cyprus | Domestic cat (*Felis catus domesticus*) | KY215817 | 7 | *H. felis* genotype I |
| Western Asia | Cyprus | Domestic cat (*Felis catus domesticus*) | KY215818 | 7 | *H. felis* genotype I |
| Western Asia | Israel | Domestic cat (*Felis catus domesticus*) | KC138531 | 13 | *H. canis* |
| Western Asia | Israel | Domestic cat (*Felis catus domesticus*) | KC138532 | 13 | *H. canis* |
| Western Asia | Israel | Domestic cat (*Felis catus domesticus*) | KC138539 | 13 | *H. canis* |
| Western Asia | Israel | Domestic cat (*Felis catus domesticus*) | KC138540 | 13 | *H. canis* |
| Western Asia | Israel | Domestic cat (*Felis catus domesticus*) | KC138542 | 13 | *H. felis* genotype I |
| Western Asia | Israel | Domestic cat (*Felis catus domesticus*) | KC138541 | 13 | *H. felis* genotype I |
| Western Asia | Israel | Domestic cat (*Felis catus domesticus*) | KC138536 | 13 | *H. felis* genotype I |
| Western Asia | Israel | Domestic cat (*Felis catus domesticus*) | KC138534 | 13 | *H. felis* genotype I |
| Western Asia | Israel | Domestic cat (*Felis catus domesticus*) | KC138533 | 13 | *H. felis* genotype I |
| Western Asia | Israel | Flea (*Ctenocephalides felis*) | MG722715 | 60 | *H. felis* genotype I |
| Western Asia | Israel | Flea (*Ctenocephalides felis*) | MG722716 | 60 | *H. felis* genotype I |
| Western Asia | Israel | Flea (*Ctenocephalides felis*) | MG722717 | 60 | *H. felis* genotype I |
| Western Asia | Israel | Flea (*Ctenocephalides felis*) | MG722718 | 60 | *H. felis* genotype I |
| Western Asia | Turkey | Tick (*Rhipicephalus turanicus,* from human) | MF383513 | 44 | *H. felis* genotype I |
| Western Asia | Turkey | Tick (*Rhipicephalus sanguineus*, from dog) | JQ867388 | 26 | *H. felis* genotype I |
| Western Asia | Turkey | Tick (*Haemaphysalis sulcata*, from human) | KF034779 | 27 | *H. felis* genotype II |
| Western Asia | Turkey | Tick (*Ixodes ricinus*, from human) | KF034780 | 27 | *H. felis* genotype II |
| Southern Europe | Bosnia and Herzegovina | European wild cat (*Felis silvestris silvestris*) | MF614156 | 41 | *H. felis* genotype I |
| Southern Europe | Bosnia and Herzegovina | European wild cat (*Felis silvestris silvestris*) | KX757033 | 40 | *H. felis* genotype II |
| Southern Europe | Bosnia and Herzegovina | European wild cat (*Felis silvestris silvestris*) | KX757032 | 40 | *H. silvestris* |
| Southern Europe | Bosnia and Herzegovina | European wild cat (*Felis silvestris silvestris*) | KX757031 | 40 | *H. silvestris* |
| Southern Europe | Bosnia and Herzegovina | European wild cat (*Felis silvestris silvestris*) | MF614155 | 41 | *H. silvestris* |
| Southern Europe | Bosnia and Herzegovina | European wild cat (*Felis silvestris silvestris*) | MF614157 | 41 | *H. martis*^e^ |
| Southern Europe | Croatia | Tick (from cat) | MH656727 | unpublished | *H. felis* genotype I |
| Southern Europe | Italy | Domestic cat (*Felis catus domesticus*) | KY649446 | 38 | *H. canis* |
| Southern Europe | Italy | Domestic cat (*Felis catus domesticus*) | KY649442 | 38 | *H. felis* genotype I |
| Southern Europe | Italy | Domestic cat (*Felis catus domesticus*) | KY649443 | 38 | *H. felis* genotype I |
| Southern Europe | Italy | Domestic cat (*Felis catus domesticus*) | KY649444 | 38 | *H. felis* genotype I |
| Southern Europe | Italy | Domestic cat (*Felis catus domesticus*) | KY511259 | 5 | *H. felis* genotype I |
| Southern Europe | Italy | Domestic cat (*Felis catus domesticus*) | KY649445 | 38 | *H. silvestris* |
| Southern Europe | Portugal | Domestic cat (*Felis catus domesticus*) | AB896686 | 48 | *H. felis* genotype I |
| Southern Europe | Portugal | Domestic cat (*Felis catus domesticus*) | AB896687 | 48 | *H. felis* genotype I |
| Southern Europe | Portugal | Domestic cat (*Felis catus domesticus*) | AB896688 | 48 | *H. felis* genotype I |
| Southern Europe | Portugal | Domestic cat (*Felis catus domesticus*) | AB896689 | 48 | *H. felis* genotype I |
| Southern Europe | Portugal | Domestic cat (*Felis catus domesticus*) | AB896690 | 48 | *H. felis* genotype I |
| Southern Europe | Portugal | Domestic cat (*Felis catus domesticus*) | AB896691 | 48 | *H. felis* genotype I |
| Southern Europe | Portugal | Domestic cat (*Felis catus domesticus*) | AB896692 | 48 | *H. felis* genotype I |
| Southern Europe | Portugal | Domestic cat (*Felis catus domesticus*) | AB896693 | 48 | *H. felis* genotype I |
| Southern Europe | Portugal | Domestic cat (*Felis catus domesticus*) | AB896694 | 48 | *H. felis* genotype I |
| Southern Europe | Portugal | Domestic cat (*Felis catus domesticus*) | AB872992 | 48 | *H. felis* genotype I |
| Southern Europe | Portugal | Domestic cat (*Felis catus domesticus*) | AB872993 | 48 | *H. felis* genotype I |
| Southern Europe | Portugal | Domestic cat (*Felis catus domesticus*) | AB872994 | 48 | *H. felis* genotype I |
| Southern Europe | Portugal | Domestic cat (*Felis catus domesticus*) | AB872995 | 48 | *H. felis* genotype I |
| Southern Europe | Portugal | Tick (*Rhipicephalus sanguineus*, from dog) | AB872945 | 47 | *H. felis* genotype I |
| Southern Europe | Portugal | Tick (*Rhipicephalus sanguineus*, from cat) | AB872946 | 47 | *H. felis* genotype I |
| Southern Europe | Portugal | Tick (*Rhipicephalus sanguineus*, from cat) | AB872947 | 47 | *H. felis* genotype I |
| Southern Europe | Portugal | Tick (*Rhipicephalus sanguineus*, from cat) | AB872948 | 47 | *H. felis* genotype I |
| Southern Europe | Spain | Domestic cat (*Felis catus domesticus*) | AY628681 | 32 | *H. felis* genotype I |
| Southern Europe | Spain | Domestic cat (*Felis catus domesticus*) | AY620232 | 32 | *H. felis* genotype I |
| Western Europe | France | Domestic cat (*Felis catus domesticus*) | EU622909 | 33 | *H. canis* |
| Western Europe | France | Domestic cat (*Felis catus domesticus*) | EU622910 | 33 | *H. canis* |
| Western Europe | Switzerland | Domestic cat (*Felis catus domesticus*) | MH078194 | 45 | *H. silvestris* |

^a^ *Hepatozoon* spp. sequences previously described as *H. felis* from lions and spotted hyenas in Zambia (Reference 59, Accession numbers KF270642-KF270644, KF270646, KF270658, KF270659,KF270663, KF270664, KF270666, KF270667, KF270669, KF270673), from ocelots in Brazil (Reference 35, Accession numbers KX776286, KX776299, KX776303, KX776307, KX776329) and from spotted hyenas in Tanzania (Reference 36, Accession number EF188809) were not included in the dataset because the amplified fragment was different from the region of the 18S rRNA gene amplified by the PCR protocol used in this study. According to reference 33, the *H. canis* sequence FJ213775 retrieved from GenBank as obtained from a cat was considered as obtained from a dog and was not included in the dataset.

^b^ BLAST analysis showed 98% of nucleotide identity with *H. silvestris* sequences from European wild cats from Bosnia and Herzegovina available in GenBank (accession numbers KX757031 and KX757032).

^c^ BLAST analysis showed 98% of nucleotide identity with several *H. felis* sequences of both *H. felis* genotype I and II available in GenBank (e.g. accession numbers JN584475 and KX757033).

^d^ the three sequences were phylogenetically grouped in the same distinct significant cluster of *Hepatozoon* spp.

^e^ BLAST analysis also showed 100% of nucleotide identity with *H. martis* sequence available in GenBank (accession numbers MG136687, MG136688, EF222257, EU686690 and KU198330).
